# Hypolipidemic Activity of *Eryngium carlinae* on Streptozotocin-Induced Diabetic Rats

**DOI:** 10.1155/2012/603501

**Published:** 2011-11-24

**Authors:** Ruth Noriega-Cisneros, Omar Ortiz-Ávila, Edgar Esquivel-Gutiérrez, Mónica Clemente-Guerrero, Salvador Manzo-Avalos, Rafael Salgado-Garciglia, Christian Cortés-Rojo, Istvan Boldogh, Alfredo Saavedra-Molina

**Affiliations:** ^1^Instituto de Investigaciones Químico-Biológicas, Universidad Michoacana de San Nicolás de Hidalgo, 58030 Morelia, Michoacán, Mexico; ^2^Department of Microbiology and Immunology, University of Texas Medical Branch at Galveston, Galveston, TX 77555, USA

## Abstract

Diabetes mellitus (DM) is a significant risk factor for the development of cardiovascular complications. This study was undertaken to investigate the effect of chronic administration of ethanolic extract of *Eryngium carlinae* on glucose, creatinine, uric acid, total cholesterol, and triglycerides levels in serum of streptozotocin- (STZ-) induced diabetic rats. Triglycerides, total cholesterol, and uric acid levels increased in serum from diabetic rats. The treatment with *E. carlinae* prevented these changes. The administration of *E. carlinae* extract reduced the levels of creatinine, uric acid, total cholesterol, and triglycerides. Thus administration of *E. carlinae* is able to reduce hyperlipidemia related to the cardiovascular risk in diabetes mellitus.

## 1. Introduction

Diabetes mellitus (DM) describes a metabolic disorder of multiple etiologies characterized by chronic hyperglycemia with disturbances of carbohydrate, fat, and protein metabolism resulting from defects in insulin secretion, insulin action, or both [1]. The effects of DM include long-term damage, dysfunction, and failure of various organs. Diabetic people present a 2–4-fold higher risk of developing cardiovascular disease than normoglycemic population of similar age and sex. The cardiovascular complications attributable to atherosclerosis are responsible for 70–80% of all causes of death in patients with diabetes and represent more than 75% of all hospitalizations for diabetic complications [2]. The primary goal of diabetes treatment is the prevention of macrovascular complications (myocardial infarction, heart failure, ischemic stroke), as well as the microvascular complications (retinopathy, neuropathy, and nephropathy); for that reason, most patients require not only a good glycemic control but also treatment for dyslipidemia [3]. Dyslipidemia (disruption in the normal levels of lipids, mainly cholesterol and triglycerides) is considered a major cardiovascular risk factor in diabetes [2]. Therefore, the detection of dyslipidemia and its treatment to reduce the cardiovascular risk and its consequences are required in diabetic patients.

The growing need to find alternatives for the treatment of diabetes justifies the study of medicinal plants used in traditional medicine. There are several reports about the beneficial effects of a wide range of plants to treat diabetes. Hypoglycemic effect was observed in extracts of plants such as *Amaranthus viridis *[4] and *Psidium guajava *[5] whose effects are almost comparable with the synthetic drug tolbutamide, while *Zaleya decandra *[6] has effects equivalent to glibenclamide. Other plants also appear to exert their beneficial effects by improving the dyslipidemia and oxidative stress that characterize diabetes. For example, *Morus indica L*. [7] decreases LDL-cholesterol and VLDL-cholesterol level and increases HDL-cholesterol levels, while *Phyllanthus amarus *[8] improves antioxidant status and reduces the risk of oxidative stress. 

In México, the use of plants for diabetes treatment is generally carried out in an empirical basis. In this regard, it seems that many plants for diabetes treatment were originally used for a variety of kidney disorders and most notably for their diuretic effect [9]. This study was conducted to analyze the effect of chronic administration of ethanolic extract of *E. carlinae* in diabetic rats. This plant has often used in traditional medicine to treat diabetes; however there are no studies indicating the effects of its consumption. *Eryngium carlinae* F. Delaroche is a perennial herb plant considered a weed belonging to the family Umbelliferae. It is distributed in forests of fir, pine and pine-oak hillsides, and canyons, deep soils rich in organic matter. It is distributed from 2020–2590 meters above sea level. It is commonly known as “Frog herb”. Decoctions of the aerial parts of the plant are used to treat coughs, indigestion, diseases of the prostate, lipid disorders, and diabetes [10]. It has been attributed healing and diuretic properties to extracts from the plant. Other species of *Eryngium* (*Eryngium columnare*) have been used to treat kidney disease, diarrhea, allergy, cough, and cancer [10].

Therefore, the aim of this research was to determine the effect of chronic oral administration of ethanolic extract of *E. carlinae* on some biochemical parameters in STZ-induced diabetic rats.

## 2. Materials and Methods

### 2.1. Vegetal Material

Plant samples of *Eryngium carlinae* F. Delaroche were collected in September and October, 2008, in the region of Nuevo San Juan Parangaricutiro, Michoacán, México. The plant was identified by Miguel Angel Bello-González PhD (Faculty of Agrobiology, Universidad Michoacana de San Nicolás de Hidalgo) genus and species preserving. A voucher specimen was deposited at the Biology Faculty Herbarium of the Universidad Michoacana de San Nicolás de Hidalgo (no. 15214). The aerial part of the plant was dried at room temperature and pulverized. 

### 2.2. Extract Preparation

Ethanolic extract of the plant was prepared by adding 1,000 mL of absolute ethanol to 100 g of plant powder and kept at 5°C for five days. The extract was then filtered, concentrated in a rotary evaporator at vacuum and at a temperature lower than 50°C, evaporated at room temperature, suspended in ethanol 96%, and stored in the dark at 5°C. The percentage yield of the dry residue was 1.27% w/w.

### 2.3. Animals

Male Wistar rats weighing 280–360 g were used. They were housed and maintained at room temperature with day/night cycles of 12 h. They were fed with standard rodent diet and water *ad libitum*. We followed the recommendations of the regulatory standard for the use of animals issued by the Mexican Ministry of Agriculture in the paragraph of the Federal Regulations for the Use and Care of Animals (NOM-062-ZOO-1999). This research was also approved by the Institutional Committee for Use of Animals of the Universidad Michoacana de San Nicolás de Hidalgo.

### 2.4. Diabetes Induction

Diabetes was induced by intraperitoneal administration of STZ (45 mg/kg of body weight) dissolved in citrate buffer (pH 4.5). Control rats were injected with citrate buffer alone. Five days after streptozotocin administration, the glucose levels were determined to confirm diabetes. Rats exhibiting blood glucose levels >300 mg/dL were considered for the study.

### 2.5. Experimental Protocol

Rats were randomly divided into four groups of six rats. Group I, control (vehicle, ethanol 50%), Group II, control + *E. carlinae* (30 mg/kg of body weight), Group III, diabetic (vehicle, ethanol 50%), Group IV, diabetic + *E. carlinae* (30 mg/kg of body weight). The extract was given by oral administration using oral gavage. The treatment was continued daily for 40 days.

### 2.6. Effects of *E. Carlinae* on Glycemia and Body Weight 

Glucose estimation was started just before extract administration and followed every 5 days using a commercial glucometer (Accu-Check Sensor III Glucometer) through a puncture in the tail tip, and animal weight was recorded at a time during the 40 days.

### 2.7. Effects of *E. Carlinae* on Hematological and Biochemical Parameters

At 40 days of treatment, the animals were fasted overnight and sacrificed by decapitation. The blood was collected, and serum was separated and used for biochemical estimations. The levels of glucose, creatinine, uric acid, total cholesterol, and triglycerides were estimated spectrophotometrically using a commercial assay kit (BioSystems, Spain). Glucose was determined measuring enzymatic oxidation catalyzed through the Trinder reaction [11]. Creatinine was determined by a kinetic method without deproteinization as reported by Junge et al. [12]. Uric acid was determined by an enzymatic photometric method using TBHBA (2, 4, 6-tribromide-3-hydroxybenzoic acid). Total cholesterol was determined measuring the enzymatic hydrolysis and oxidation with the Trinder reaction [13]. Triglycerides were determined by an enzymatic colorimetric method using glycerol-3-phosphate oxidase [14]. Whole blood samples were used for hemoglobin (Golden Bell, México) and glycosylated hemoglobin (DiaSys, Germany) determinations using commercial assay kits.

### 2.8. Statistical Analysis

The results were expressed as the mean ± Standard Error (SE) of at least six independent experiments. Statistical significances (*P* ≤ 0.05) were determined with Student's *t*-test using GraphPad Prism 5 software.

## 3. Results

Changes in the body weight of the rats are presented in Table 1. In control groups, the extract did not alter the body weight gain as this parameter increased 31% and 29% at the end of the treatment in both control and control +* E. carlinae* groups, respectively, compared to their initial body weight. Similarly, no differences in body weight were detected between diabetic and diabetic + *E. carlinae* groups throughout the study.

With regard to blood glucose levels, Table 1 shows the records taken during the 40 days of treatment with *E. carlinae*. It can be observed a significant increase in glucose levels in diabetic groups compared with controls at the beginning of treatment. Oral administration of *E. carlinae* (30 mg/kg of body weight) for 40 days showed no reduction in blood glucose.

Table 2 shows the levels of hemoglobin and glycosylated hemoglobin in the different groups. The results show that hemoglobin levels are not affected by treatment with *E. carlinae*. Glycosylated hemoglobin determinations show a significant increase in the percentage of glycosylated hemoglobin in diabetic group compared to control group, and the treatment with *E. carlinae* did not change this behavior.

Table 3 shows the levels of glucose, creatinine, uric acid, total cholesterol, and triglycerides in serum of all groups. It is important to point out that these determinations were made with animals 12 h fast. The levels of glucose, uric acid, total cholesterol, and triglycerides in serum were significantly increased in the diabetic group when compared to control group. The treatment with *E. carlinae* decreased creatinine, uric acid, total cholesterol, and triglycerides levels in serum compared with the diabetic group.

## 4. Discussion and Conclusions

Oral administration of the ethanolic extract of *E. carlinae* was conducted for 40 days in a concentration of 30 mg/kg of body weight. During the treatment it was observed that the control group had a weight gain corresponding to age-appropriate growth (Table 1). In contrast, the diabetic group showed no weight gain, which is characteristic of this model of diabetes, and the treatment with *E. carlinae* did not change this tendency. 

Glycemic control is a priority in diabetic patients because it has relationship with a decrease in microvascular complications in diabetes [15]. In the measurements made at the end of treatment (Table 1), there was no reduction in glucose level in the groups that received *E. carlinae*. The glycohemoglobin (HbA1) is a general term used to describe hemoglobin that has been modified by the addition of glucose through a nonenzymatic mechanism, and the HbA1c is one of those glycosides compounds in particular that reflects average blood glucose in patient 2 or 3 months before blood collection [16, 17]. According to the United Kingdom Prospective Diabetes Study (UKPDS), each 1% reduction in glycosylated hemoglobin (HbA1c) was associated with a 37% reduction in microvascular complications, 18% fewer myocardial infarction, and 21% fewer diabetes-related deaths [15]. The results obtained from the measurement of HbA1c (Table 2) show a clear difference between control (HbA1c 3.32%) and diabetic (HbA1c 7.48%) group. The diabetic group that received *E. carlinae* did not show significant decrease in HbA1c (HbA1c 7.34%). Based on this and the results from Table 1, we can conclude that the ethanolic extract *E. carlinae* has not hypoglycemic effect. However, it is noteworthy that many of the plants that have been reported as hypoglycemic agents are less effective in severe diabetes [18], so it may be necessary to evaluate the extract in another model of diabetes to rule out completely its possible hypoglycemic action.

Diabetes leads to renal dysfunction [19]. Measurement of creatinine can be considered as a marker of renal dysfunction. Our results suggest that the administration of *E. carlinae* in the diabetic group decreased level of creatinine (Table 3), probably by improving renal function. The complex pathogenesis for the development of diabetic nephropathy is not well understood. One factor that has been associated with renal and cardiovascular disease is serum uric acid. It has been found that level of uric acid circulating in the upper end of normal range concentration is an independent predictor for development of diabetic nephropathy, which supports the concept that uric acid may be involved in the pathogenesis of diabetic microvascular complications [20]. Uric acid level in the diabetic group is elevated compared to control group (Table 3). Interestingly, the higher levels of uric acid in diabetic rats are diminished significantly by the administration of *E. carlinae*, which could be related to a delay in the onset of the complications of diabetes, as has been previously suggested [20]. Abnormalities in the metabolism of lipids are one of the most frequent complications in diabetes. Among the most common lipid abnormalities is the elevation of cholesterol and triglycerides. Hypertriglyceridemia has been identified as a major risk factor for cardiovascular complications [21]. Diabetic group had elevated triglycerides level compared to control group (Table 3). This increase was normalized by treatment with *E. carlinae*. With regard to total cholesterol level, the treatment with *E. carlinae* was able to reduce this level (Table 3). Based on our results we conclude that the ethanolic extract of *E. carlinae* has no hypoglycemic effect; however, by its hypolipidemic effect it could be used as an adjuvant in the treatment of diabetes. Further studies need to be done to characterize the active components of the ethanolic extract of *E. carlinae* and its mechanism of action. 

## Figures and Tables

**Table 1:** Effect of *E. carlinae* on body weight and blood glucose.

Groups	Body weight (g)		Blood glucose (mg/dL)								
	Initial	Final	Day								
	(day 0)	(day 40)	0	5	10	15	20	25	30	35	40
Control	349 ± 4	456 ± 10	102 ± 4	109 ± 13	92 ± 4	89 ± 1	93 ± 13	86 ± 4	90 ± 17	74 ± 0.0	82 ± 4
Control *+ E. carlinae *	317 ± 12	408 ± 17	11 ± 7	116 ± 10	97 ± 4	95 ± 2	105 ± 2	90 ± 4	94 ± 4	84 ± 3	85 ± 2
Diabetic	307 ± 15	305 ± 17	551 ± 20*	423 ± 16	411 ± 13	549 ± 21	535 ± 22	580 ± 15	538 ± 22	530 ± 30	571 ± 29
Diabetic + *E. carlinae *	298 ± 12	302 ± 14	436 ± 16*	372 ± 14	365 ± 19	475 ± 33	373 ± 24	410 ± 29	405 ± 38	566 ± 15	537 ± 27

Values are mean ± SE. *P ≤ 0.05 versus control group day 0.

**Table 2:** Effect of *E. carlinae* on hemoglobin and glycosylated hemoglobin (HbA1c).

Groups	Hemoglobin (mg/dL)	Glycosylated hemoglobin (% HbA1c)
Control	16 ± 0.7	3.32 ± 0.07
Control + *E. carlinae *	16 ± 0.1	ND
Diabetic	17 ± 0.5	7.48 ± 0.08*
Diabetic + *E. carlinae *	16 ± 0.8	7.34 ± 0.04

Values are mean ± SE. *P ≤ 0.05 versus control group.

**Table 3:** Effect of *E. carlinae* on biochemical parameters after oral administration during 40 days.

Groups	Glucose (mg/dL)	Creatinine (mg/dL)	Uric acid (mg/dL)	Total cholesterol (mg/dL)	Triglycerides (mg/dL)
Control	85 ± 6	0.59 ± 0.02	1.40 ± 0.04	59 ± 3	153 ± 18
Control + *E. carlinae *	79 ± 3	0.56 ± 0.01	1.17 ± 0.26	54 ± 3	128 ± 7
Diabetic	359 ± 8*	0.48 ± 0.06	2.43 ± 0.50*	78 ± 6*	252 ± 19*
Diabetic + *E. carlinae *	376 ± 21	0.32 ± 0.02**^+^**	1.10 ± 0.33**^+^**	46 ± 6**^+^**	115 ± 7**^+^**

Values are mean ± SE of at least *n* = 6. *P ≤ 0.05 versus control group; **^+^** *P* ≤ 0.05 versus diabetic group.
